# The superiority of PMFs on reversing drug resistance of colon cancer and the effect on aerobic glycolysis-ROS-autophagy signaling axis

**DOI:** 10.32604/or.2024.048778

**Published:** 2024-11-13

**Authors:** YUQIN YIN, YU WU, HONGLIANG HUANG, YINGYING DUAN, ZHONGWEN YUAN, LIHUI CAO, JINJIN YING, YONGHENG ZHOU, SENLING FENG

**Affiliations:** 1 Department of Pharmacy, Guangdong Provincial Key Laboratory of Major Obstetric Diseases, Guangdong Provincial Clinical Research Center for Obstetrics and Gynecology, The Third Affiliated Hospital of Guangzhou Medical University, Guangzhou, 510150, China; 2 School of Pharmaceutical Sciences, Guangzhou Medical University, Guangzhou, 511436, China; 3 Department of Pharmacy, The Fourth Affiliated Hospital of Guangzhou Medical University, Guangzhou, 511300, China

**Keywords:** Polymethoxylated flavones (PMFs), Colon cancer, Drug resistance, Aerobic glycolysis-ROS-autophagy, Warburg effect

## Abstract

**Background:**

Polymethoxylated flavones (PMFs) are compounds present in citrus peels and other Rutaceae plants, which exhibit diverse biological activities, including robust antitumor and antioxidant effects. However, the mechanism of PMFs in reversing drug resistance to colon cancer remains unknown. In the present study, we aimed to investigate the potential connection between the aerobic glycolysis-ROS-autophagy signaling axis and the reversal of PTX resistance in colon cancer by PMFs.

**Methods:**

MTT Cell viability assay and colony formation assay were used to investigate the effect of PMFs combined with PTX in reversing HCT8/T cell resistance *ex vivo*; the mRNA and protein levels of the target were detected by SDS-PAGE (sodium dodecyl sulfate-polyacrylamide gel electrophoresis), quantitative real-time fluorescence polymerase chain reaction (qRT-PCR) and Western blot protein immunoblotting (WB); An HCT8/T cell xenograft model was established to investigate the MDR reversal activity of PMFs *in vivo*; The extracellular acidification rate (ECAR) and the oxygen consumption rate (OCR) were detected to assess the cellular oxygen consumption rate and glycolytic process.

**Results:**

HCT8/T cells demonstrated significant resistance to PTX, up-regulating the expression levels of ABCB1 mRNA, P-gp, LC3-I, and LC3-II protein, and increasing intracellular reactive oxygen species (ROS) content. PMFs mainly contain two active ingredients, nobiletin, and tangeretin, which were able to reverse drug resistance in HCT8/T cells in a concentration-dependent manner. PMFs exhibited high tolerance in the HCT8/T nude mouse model while increasing the sensitivity of PTX-resistant cells and suppressing tumor growth significantly. PMFs combined with PTX reduced extracellular acidification rate (ECAR) and oxygen consumption rate (OCR) in HCT8/T cells. Additionally, PMFs reduced intracellular ROS content, down-regulated the expression levels of autophagy-related proteins LC3-I, LC3-II, Beclin1, and ATG7, and significantly reduced the number of autophagosomes in HCT8/T cells.

**Conclusions:**

The present study demonstrated that PMFs could potentially reverse PTX resistance in colon cancer by regulating the aerobic glycolysis-ROS-autophagy signaling axis, which indicated that PMFs would be potential potentiators for future chemotherapeutic agents in colon cancer.

## Introduction

Colon cancer is the second most diagnosed cancer globally, which is a primary cause of cancer-related deaths [1]. Chemotherapy is the primary treatment for clinically advanced colon cancer, but limitations have been discovered in current treatments due to the emergence of multidrug resistance (MDR) in tumor cells [2].

Paclitaxel (PTX), a commonly used chemotherapy drug in clinical, contains the role of arresting the cell cycle into the G2/M phase and promoting apoptosis and has been used to treat multiple types of cancer from ovarian, breast, lung, and pancreatic [3]. However, PTX contains physicochemical properties of poor water solubility, the bioavailability of intravenously administered PTX is low and causes a variety of adverse side effects including cardiovascular toxicity, gastrointestinal side effects, and hypersensitivity reactions. P-gp is present abundantly in the colon, when PTX is administered orally, low bioavailability is also observed owing to the drug efflux of P-gp [4]. Due to these limitations, PTX has not been used in standard chemotherapy for colon cancer. However, increasing data implicate that new therapeutic approaches with PTX may be effective in treating colon cancer. Currently, there are three lines of research to improve the bioavailability and reduce the side effects of PTX, including the development of PTX conjugates or prodrugs to overcome the limitation of aqueous solubility, the use of PTX in combination with P-gp substrates or inhibitors to improve bioavailability, and the use of targeted and nano-sized delivery systems to deliver PTX directly to the tumor site [4]. PTX demonstrated inhibitory effects against colon cancer in both *ex vivo* and *in vivo* experiments when combined with other P-gp inhibitors, such as itraconazole [5] and BEZ235 [6]. These researches indicate that PTX would be a potential therapeutic option for colon cancer.

Acquired multidrug resistance (MDR) in colon cancer is associated with a variety of factors, and the overexpression of ATP-binding cassette subfamily B member 1 (ABCB1/P-gp) has been shown to be the crucial regulator of cancer progression [7]. Due to the overexpression of the ABCB1 drug efflux pump, common chemotherapeutic drugs can be excreted by P-gp, leading to decreased chemotherapeutic efficacy and high drug resistance in colon cancer [8]. The formation of MDR is also connected to reactive oxygen species (ROS) generation and autophagy. High levels of ROS are believed to be a characteristic of drug-resistant cancer cells. Tumor cells can remain active under oxidative stress, acquiring drug resistance via avoiding apoptosis [9]. Increased ROS production is regarded as a key factor in P-glycoprotein (P-gp) overexpression in tumor cells [10]. The role of autophagy in tumor drug resistance exhibits two sides: autophagy eliminates damaged organelles to avoid cell death, while on the other side, excessive autophagy induces programmed tumor cell death [11]. However, autophagy in advanced colon cancer tissues correlates positively with malignant progression and cellular drug resistance acquisition [12]. Previous research has demonstrated that ROS could accelerate the conversion from apoptosis to autophagy, causing cancer cells to become drug-resistant [13]. Therefore, it is possible to reverse MDR formation and improve chemotherapy efficiency through the ROS-autophagy signaling axis.

Warburg effect is also strongly associated with the development of drug resistance in tumors. It is considered one of the hallmarks of cancer, referring to the reprogramming of cancer cells through energy metabolism, which tends to derive energy through aerobic glycolysis rather than oxidative phosphorylation. As an anaerobic metabolic process, though glycolysis generates ATP less efficiently, it contributes to tumor growth by responding to the energy needs quickly and creating an acidic microenvironment [14]. Increasing the Warburg effect results in higher levels of ATP, which activate ATP-dependent transport proteins that promote drug resistance [15]. Overexpression of genes related to glycolysis in colon cancer tissues has been observed to enhance cellular aerobic glycolysis and inhibit the tricarboxylic acid cycle [16]. In addition, the use of glycolysis inhibitors has been observed to inhibit cellular metabolism and increase the sensitivity of tumor cells to PTX, thereby reducing drug resistance of tumor cells [17]. These findings indicated that inhibiting aerobic glycolysis may reverse drug resistance in colon cancer.

Polymethoxylated flavones (PMFs) are flavonoids that possess potent anti-inflammatory and antitumor properties, which have inhibitory effects on various cancers from the colon, breast, and lung [18]. The main mechanisms involve inhibiting tumor cell proliferation and enhancing the sensitivity of chemotherapeutic drugs [19]. In our prior research, nobiletin, which is a compound in PMFs, exhibited the ability to enhance the antitumor effect of PTX in MDR cancer cells by inhibiting P-gp [20]. Since nobiletin is the most abundant substance in PMFs [21], we speculated that PMFs may have similar activity in reversing tumor PTX resistance and have the potential to become an ideal P-gp inhibitor in the future.

In this study, we aimed to investigate the role of PMFs in reversing PTX resistance in colon cancer and to analyze the effects on the aerobic glycolysis-ROS-autophagy signaling axis. The results of this study may provide new inspiration for future research directions.

## Materials and Methods

### Quantitative analysis of the main components of PMFs

Nobiletin (MB6570-S, purity >98%), Tangeretin (MB5320-S, purity >98%), Hesperidin (MB5810, purity >98%), paclitaxel (M0812A, purity >99.0%, PTX) were purchased from Dalian Meilun Biotechnology Co. Ltd. (China). Chenpi extract (*Citrus aurantium* L., CP), Zhishi extract (*Citrus aurantium* L., ZS), 30%/60%/95% PMFs were obtained from Guizhou HYT-BlueF Co., Ltd. (China). The Agilent 1260 HPLC system was used to establish the quantitative analysis of the main components of PMFs. The following chromatographic conditions: Diamonsil, C18 column (250 mm × 4.6 nm × 5 μm); Temperature of column: 40°C; Mobile Phases: Methanol: 43%; Acetonitrile: 40%; Water: 17% (v/v/v); Flow rate: 1 mL/min; Injection volume: 10 μL. The absorption wavelength of tangeretin and nobiletin was 322 and 331 nm, respectively, which was used for the quality control of PMFs and the determination of the content of biological matrices such as cells, tissues, and serum.

### Cell culture and treatment

Human colorectal adenocarcinoma HCT8 and its drug-resistant cell line HCT8/T were kindly provided by Professor Zhi-Hong Jiang (Macau University of Science and Technology, Macau, China). These cells have been used steadily in our research group and were not contaminated by other cell lines during the induction process [22,23]. HCT8 and HCT8/T cells were cultured in the Roswell Park Memorial Institute 1640 (RPMI 1640) culture medium. Cells were supplemented with 10% heat-inactivated Uruguayan fetal bovine serum Fetal Bovine Serum (Prime) (excel Biologics, FSP500), 100 units/mL penicillin, and 100 g/mL streptomycin (HyClone, SV30010-1). 0.94 μM of PTX was also added to maintain the drug resistance of HCT8/T cells. Cultured cells were grown at 37°C in a humidified atmosphere of 5% CO_2_ and were passaged 2–3 times a week.

### MTT cell viability assay

HCT8 and HCT8/T cells were seeded in 96-well plates at a concentration of 5000 cells/well. After cell seeding and incubated for 24 h at 37°C under humidified conditions of 5% CO_2_, the cells were transferred to a culture medium containing PTX, DMSO, and 10 µg/mL of varying drugs (CP 10:1, ZS 10:1, 95% hesperidin, 95% PMFs, 60% PMFs, 30% PMFs) and were incubated for an extra 72 h. 10 μL of MTT reagent was then added and the cells were incubated for 4 h. The absorbance was measured using a plate reader (Spectra MAX 250; Molecular Devices, Sunnyvale, CA, USA) at 570 nm according to the manufacturing institutions.

### Colony formation assay

HCT8/T cells were seeded into 6-well plates at a density of 800 cells/well. The cells were treated with 0.94 μM PTX alone or combined with 10 µg/mL of varying extracts (CP 10:1, ZS 10:1, 95% hesperidin, 95% PMFs, 60% PMFs, 30% PMFs) for 10 days. After removing the supernatant and washing cells with PBS (Procell, PB180327), the cells were fixed with glutaraldehyde (6.0% v/v) and stained with 1% crystalline violet (0.5% w/v) for 30 min, then rinsed with sterile distilled water to remove the excess crystalline violet solution. The colonies in each well were counted and the number was analyzed using the software of Quantity one-Colony counting (BIO-RAD, California, USA).

### SDS-PAGE and Western blot analysis

HCT8 and HCT8/T cells were collected and rinsed twice with ice-cold PBS buffer, then the cells were lysed with ice-cold RIPA buffer (1M Tris-HCl, 4% SDS, 20% glycerol, 0.2% 2-mercaptoethanol) containing 1% protease inhibitor in ice for 30 min. After centrifugation for 15 min at 4°C (12,000 rpm), collecting protein in the supernatant and used for protein concentration determined by a BCA kit (P0009, Shanghai Biyuntian Biological Co., Ltd., China). For western blotting, equal amounts of proteins (10 µg) were separated using sodium dodecyl sulfate-polyacrylamide gel electrophoresis (SDS-PAGE) and then transferred to polyvinylidene fluoride (PVDF, Millipore, Darmstadt, Germany) membranes. After blocking with 5% skim milk (Nestle Carnation, New Zealand) in tris-buffered saline containing 0.1% of Tween20 (TBST) for 1 h at room temperature, the membranes were incubated overnight with primary antibodies against p-gp (diluted at 1:200), LC3 (diluted at 1:1000), Beclin (diluted at 1:1000), Atg7 (diluted at 1:1000), and β-actin (diluted at 1:3000) (Bioworld Technology, Inc., China) at 4°C. Then incubated with secondary antibodies for 1 h at room temperature and detected using the ECL Western blotting analysis system (Thermo Scientific™ Chemi-luminescent Substrate, USA).

### Reactive oxygen species (ROS) assay using flow cytometer

ROS levels were detected with the fluorescent probe 2′,7′-dichlorofluorescein diacetate (DCFH-DA, Invitrogen, Carlsbad, CA, USA). 3 mL PBS pre-cooled at 4°C was added to the suspended cells, and after centrifuging at 1500 rpm for 5 min, discard the supernatant and shake well, then remove 10 µL for cell count on a blood cell counter to adjust the cell number to 1 × 10^6^/mL. DCFH-DA was diluted 1:1000 with serum-free medium to a final concentration of 10 µM. After incubating in a cell incubator at 37°C for 20 min, the mixture was inverted every 3–5 min to ensure the probe was in complete contact with the cells. The excitation wavelength of 488 nm and the emission wavelength of 525 nm were used for flow cytometry (BDFACSAria, BD Biosciences, San Jose, CA, USA). FlowJo software was used to analyze the ROS levels of cells.

### Cellular autophagy analysis

HCT8/T cellular autophagy analysis was performed by fluorescence microscopy (Leica DM2500, Wetzlar, Germany) to observe the formation of intracellular LC3 (Bioworld Technology, Inc., China) autophagosome after the treatment of 95% PMFs (20 µg/mL) combined with PTX (0.94 µM). In the experiment, 4′, 6-diamidino-2-phenylindole (DAPI, D9542, Sigma-Aldrich) was used for nuclear staining.

### Analysis of cellular oxygen consumption rate and glycolytic process

The extracellular acidification rate (ECAR) of HCT8 and HCT8/T cells was measured by Seahorse Cell Energy Metabolism Analyzer (Agilent Seahorse XF Pro Analyzer, Agilent Technologies Inc., China) according to the manufacturer’s instructions. The oxygen consumption rate (OCR) was detected by the Seahorse XF Cell Mito Stress Test Kit with Seahorse XF96 analyzer. Similarly, the Seahorse XF Glycolytic Rate Assay Kit was used to detect the extracellular acidification rate (ECAR) and the Seahorse XF Real-Time ATP Rate Assay kit was used to detect the real-time ATP production rates from glycolysis. HCT8 and HCT8/T were seeded in an XF24-well plate (Seahorse Bioscience) at a density of 3 × 10^5^ and then allowed to attach overnight. After 48 h treatment of 95% PMFs (20 µg/mL), OCR was assessed using a sequential injection of 1 μM oligomycin, 1 μM carbonyl cyanide 4-(trifluoromethoxy) phenylhydrazone (FCCP), 1 μM antimycin and rotenone. To assess ECAR, cells were incubated with an unbuffered medium followed by injection of 10 mM glucose, 1 μM oligomycin (Sigma-Aldrich), and 80 mM 2-deoxyglucose (2-DG). The basal glycolytic capacity, the maximum glycolytic capacity of the cells was obtained from the cell energy metabolic chart.

### Quantitative real-time polymerase chain reaction (qRT-PCR)

The total RNA of HCT8 and HCT8/T cells was extracted using TRIzol reagent (Thermo Fisher Scientific, USA). RNA purity and concentration were determined using NanoDrop 2000 (Thermo Fisher Scientific, USA). The PrimeScript™ RT reagent Kit (Takara, Japan) was used to reverse transcribe 1 μg RNA according to the manufacturer’s protocol. The qPCR reaction was performed using SYBR Green Master Mix (Takara, Japan) with the procedure as follows: 95°C, 10 min, 95°C, 5 s, 40 cycles; 60°C, 30 s. The relative expression is calculated using the 2^−ΔΔCt^ formula. The ABCB1 primer sequences refer to previous literature [24].

### In vivo xenograft model

To build up the xenograft model, HCT8/T cells (2 × 10^6^ cells/mL) were injected subcutaneously at the flank near the armpits of BALB/c nude mice (female, 4 weeks old). Experiments involving animals were approved by the ethics committee of the Third Affiliated Hospital of Guangzhou Medical University (No. 80, 2023). The animals were group-housed in a plastic cage with a laminar airflow room at a temperature of 25°C and relative humidity of 60% throughout the experiment. Nude mice were acclimated for 7 days before the study. After the tumor size reached approximately 100 mm^3^, the mice were randomly divided into six groups (5 for each group): Control group; PTX group (15 mg/kg, referring to the results of the previous study [20]); PTX combined with 25 mg/kg CP10:1 group; PTX combined with 25 mg/kg ZS 10:1 group; PTX combined with 25 mg/kg Hesperidin group; PTX combined with 25 mg/kg 95% PMFs group. 5 mice in each group were administered intraperitoneally (i.p.) every 2 days for a total of 24 days. Before each administration, the tumor length (L) and width (W) were measured with vernier calipers, and the body weight and survival status of mice were recorded. At the end of the experiments, tumor tissues were excised and weighed immediately after sacrificing mice. The blood samples were also collected and stored at −80°C for further analysis. The tumor volume was calculated according to the formula: volume = (width^2^ × length)/2.

### Statistical analysis

The results of multiple experiments were presented as mean ± standard deviation (SD). All experiments were carried out at least 3 times (n = 3). GraphPad Prism 9 software was used to conduct statistical analysis. Statistically significant differences were calculated using Student’s *t*-test or one-way analysis of variance (ANOVA). *p < 0.05; **p < 0.01; ***p < 0.001 was considered statistically significant.

## Results

### The drug resistance characteristics of HCT8/T cells

**Figure 1:**
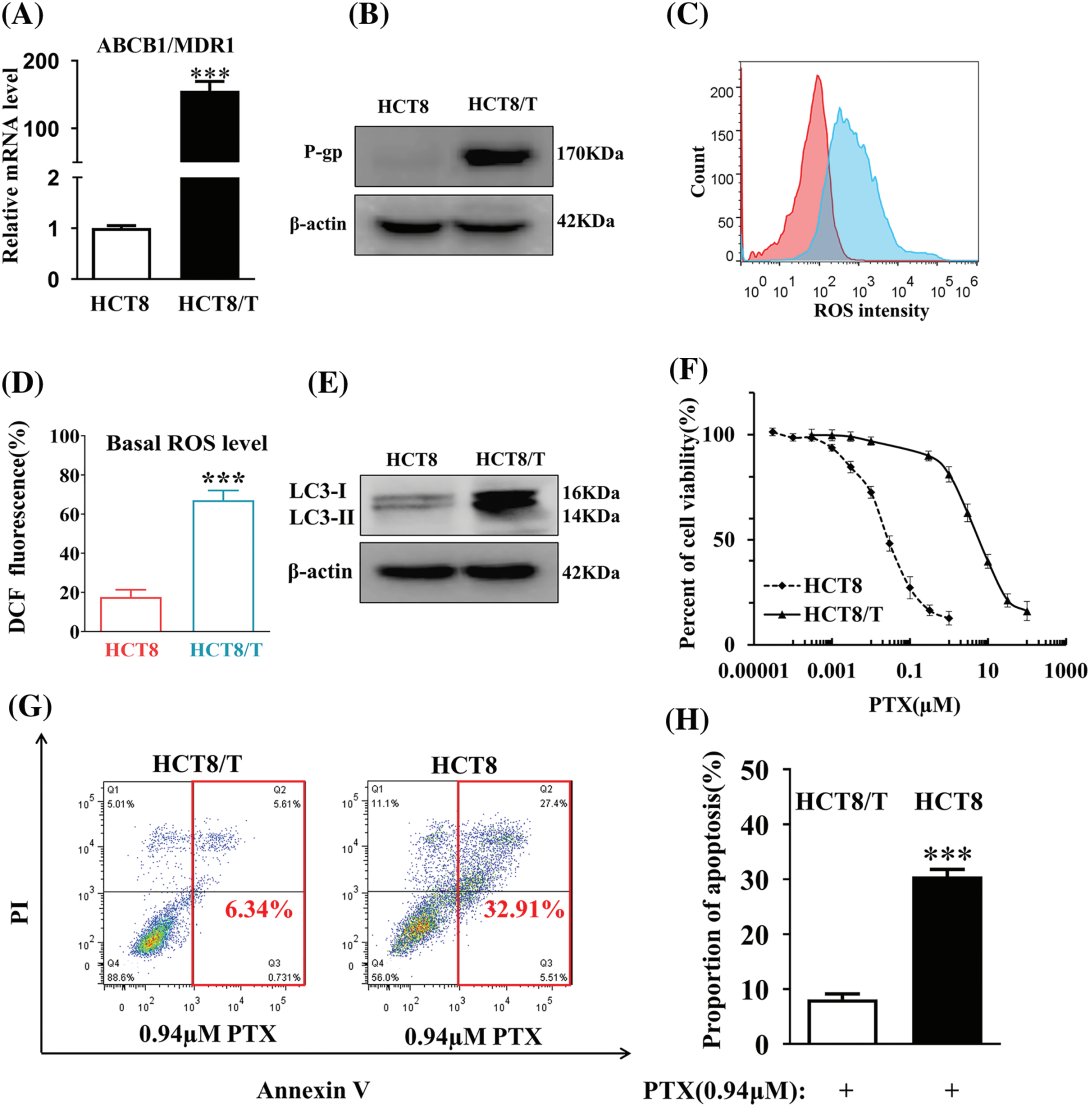
HCT8/T cells exhibit high resistance to drugs. (A) The levels of ABCB1 mRNA were measured by qRT-PCR. (B) The levels of P-gp protein in HCT8/T and HCT8 cells were assayed by Western blot. (C) MTT assay to determine the drug resistance of PTX in HCT8/T cells. (D) The level of ROS of HCT8/T and HCT8 cells was determined by FACS Aria flow cytometry. (E) The expression of LC3-I, LC3-II and β-actin protein was measured by Western blot. (F) The statistical analysis of the level of ROS in HCT8/T cells. (G) The Annexin V Apoptosis experiment of HCT8 and HCT8/T cells. (H) The proportion of apoptosis in HCT8 and HCT8/T cells (***p < 0.001).

High expression of p-glycoprotein (P-gp), ROS, and autophagosome production are closely related to the acquisition of drug resistance in tumor cells. We first analyzed the changes in ABCB1 mRNA and P-gp protein levels in HCT8/T cells by qRT-PCR and Western blot experiments. Compared to HCT8-sensitive cells, ABCB1 mRNA and P-gp protein levels were significantly higher in HCT8/T cells (Figs. 1A and 1B). Flow cytometry and Western blot results demonstrated that HCT8/T cells contained higher levels of ROS (Figs. 1C and 1D) and increased expression levels of autophagy-associated proteins LC3-I and LC3-II and glycolysis-associated protein β-actin (Fig. 1E) compared with sensitive HCT8 cells.

The viability of HCT8/T cells was determined by MTT assay. The IC50 values of HCT8 and HCT8/T cells were 0.041 and 6.21, respectively, and HCT8/T cells exhibit a multiplicity of resistance to PTX of 151.46 (Fig. 1F). The proportion of PTX-induced apoptosis in HCT8/T cells decreased compared to HCT8 cells (Figs. 1G and 1H), indicating that the drug resistance of HCT8/T was significantly increased compared with sensitive cells. This demonstrated that the HCT8/T cells did have a higher drug-resistant level compared to the sensitive cells.

### PMFs reverse HCT8/T resistance

**Figure 2:**
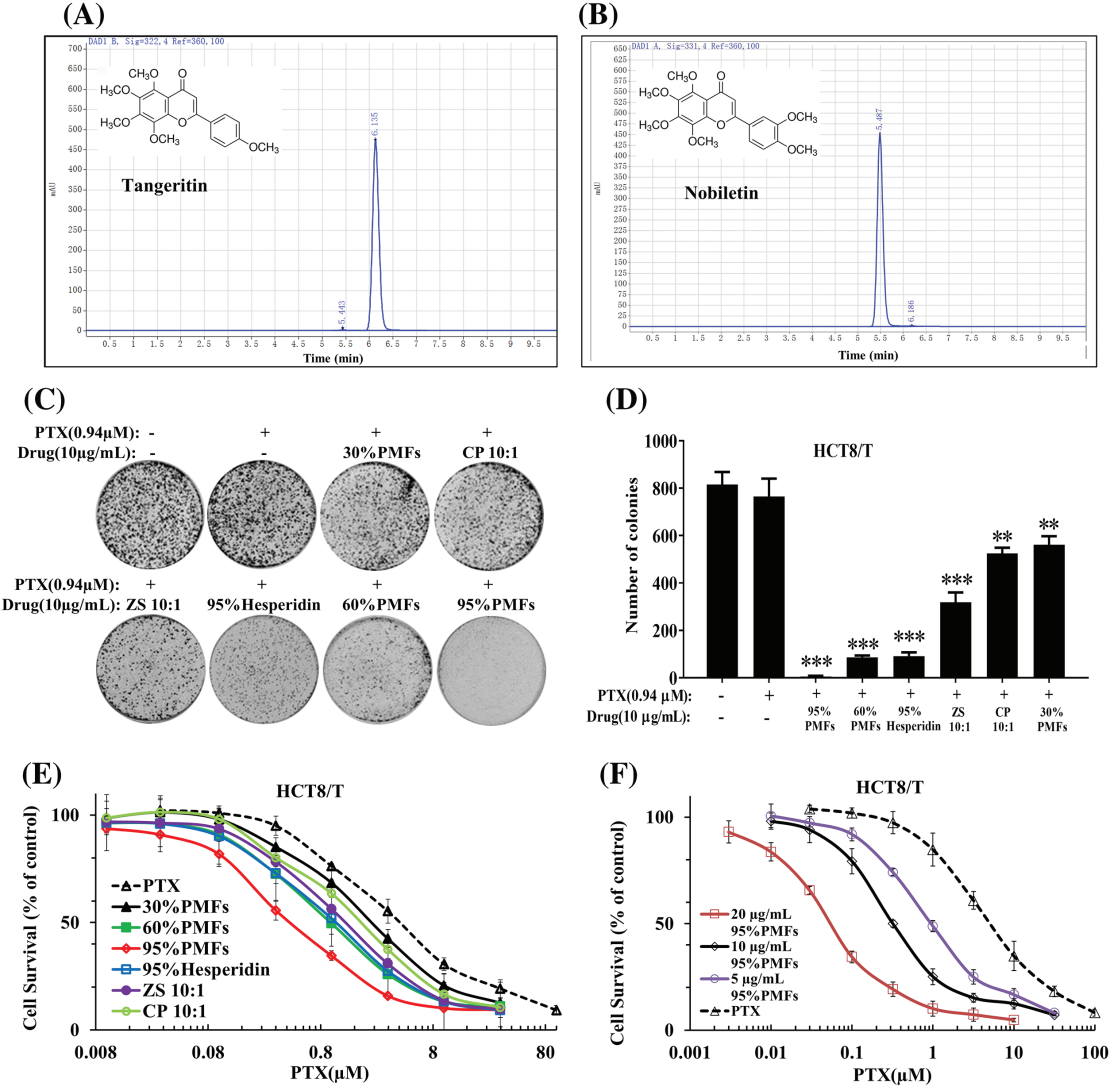
PMFs are capable of reversing HCT8/T resistance. (A, B) The residence times of Nobiletin and Tangeratin measured by Agilent 1260 HPLC system; (C) The colony formation assay of each extract; (D) The statistical analysis of colony formation in HCT8/T cells; (E) The effect of different extracts in reversing drug resistance at the same dose; (F) The effect of varying concentrations of 95% PMFs combined with PTX in reversing HCT8/T cell resistance (**p < 0.01, ***p < 0.001).

To confirm the role of PMFs in reversing HCT8/T resistance, we first analyzed the indicator components contained in the extracts with different PMFs contents for quality control and concentration determination using the HPLC system. The residence times of the indicator components in PMFs were 6.135 and 5.487 min, indicating that each extract of PMFs contained tangeretin and nobiletin (Figs. 2A and 2B).

Next, we analyzed the effect of PTX combined with different PMFs extracts to reverse drug resistance by colony formation assay. 95% PMFs combined with PTX exhibited the best inhibition with a significant reduction in cell colony density (Figs. 2C and 2D). To investigate the effect of different extracts in reversing drug resistance, each group of extracts was co-treated with PTX at the same dose (10 µg/mL) for HCT8/T cells. 30% PMFs, CP 10:1 (Chen Pi, Orange peel), ZS 10:1 (Zhi Shi, *Citrus aurantium* L.), 60% PMFs, 95% hesperidin, 95% PMFs, extracts corresponded to the reversal folds of 2.09, 2.67, 4.71, 5.03, 6.33, and 13.15, respectively (Fig. 2E). 95% PMFs showed the most efficient reversal effect of drug resistance.

In addition, we further investigated whether PMFs reverse HCT8/T resistance in a concentration-dependent manner. The 95% PMFs with the best reversal of drug resistance were selected as the subjects, treating HCT8/T cells with varying concentrations (5, 10, and 20 µg/mL) combined with PTX. The reversal folds of different concentrations of 95% PMFs were 4.92, 13.38, and 87.00, respectively (Fig. 2F). Furthermore, the *in vitro* results of our previous experiments demonstrated that PMFs showed no direct inhibitory effect toward tumor cell growth. Therefore, PMFs themselves do not interfere with the experimental results [20,22]. These results demonstrated that PMFs are effective in reversing HCT8/T resistance.

### PMFs enhance tumor sensitivity to PTX in the HCT8/T nude mouse xenograft model

**Figure 3:**
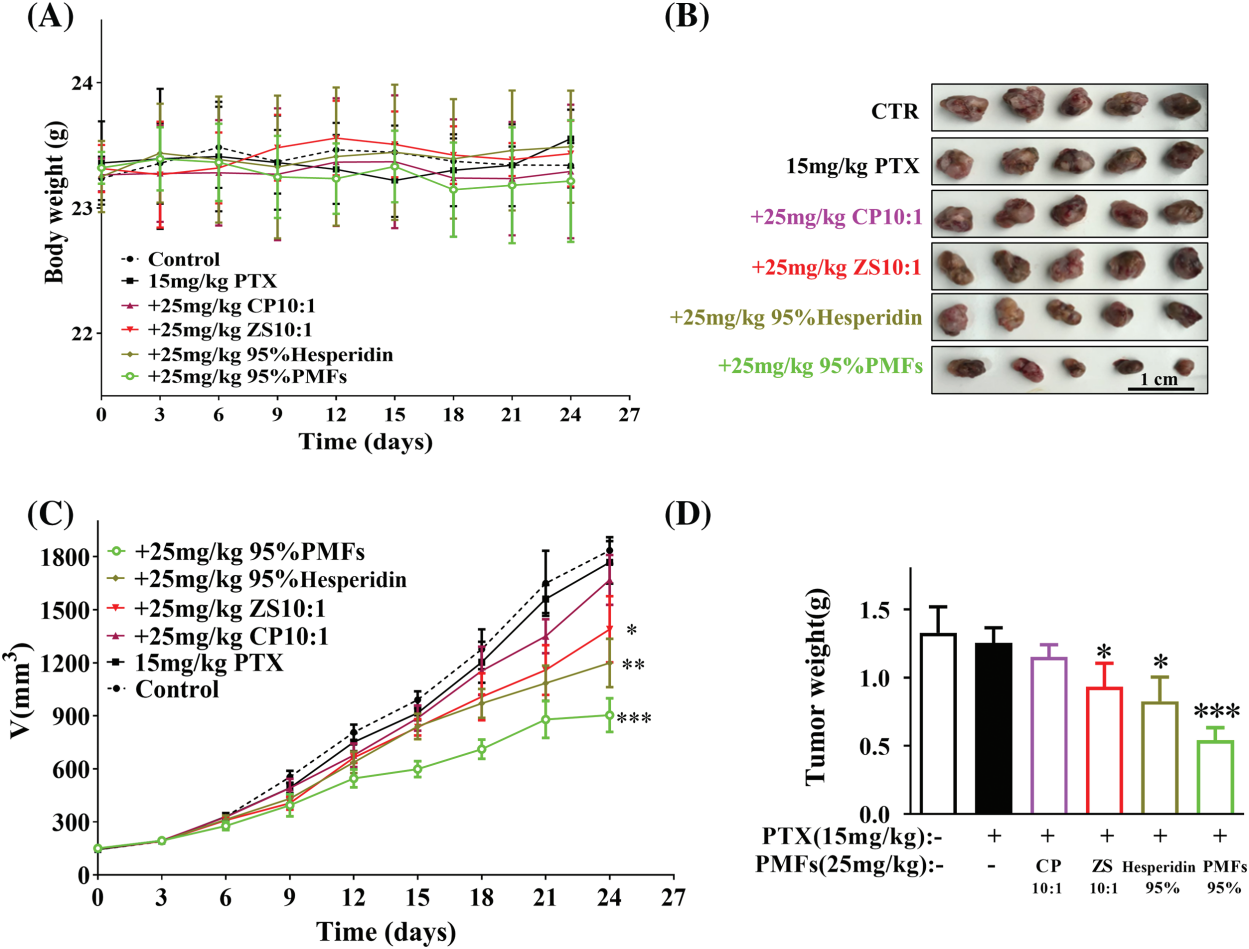
PMFs enhance tumor sensitivity to PTX in the HCT8/T nude mouse xenograft model. The body weight and tumor volume changes in each group were dynamically monitored during the experiment, and the tumor tissues were removed, photographed, and weighed at the end of the experiment. (A) Body weight of nude mice (g); (B) pictures of tumors in each group; (C) the growth curve of tumor volume (mm^3^) with time in each group; (D) the weight of tumors (g) (*p < 0.05, **p < 0.01, ***p < 0.001).

To investigate the sensitizing impact of PMFs on PTX-resistant tumors, we established the HCT8/T nude mouse model. The mice exhibited tolerance to the extracts containing PMFs, and no weight loss was observed in the PTX treatment groups (Fig. 3A). All PTX treatment groups inhibited tumor growth compared with the vector group. Among them, 95% PMFs showed the most effective anti-tumor effect, PTX-resistant tumors grew slower and decreased significantly in size (Fig. 3B). At the end of the experiment, the tumor tissues of each group were removed and weighed (Figs. 3C and 3D). Similar to the results recorded for changes in tumor volume, all PTX treatment groups showed inhibition and effective control of tumor weights. Among these groups, 95% PMFs group had the least tumor weight, which decreased by about 50% compared to the PTX group and the other treatment groups. Furthermore, our previous *in vivo* experiments showed that PMFs did not directly inhibit tumor cell growth [20,22]. All results indicated that PMFs reversed drug resistance *in vivo* while increasing tumor sensitivity to PTX and inhibiting tumor growth.

### PMFs inhibit the aerobic glycolytic process in HCT8/T cells

**Figure 4:**
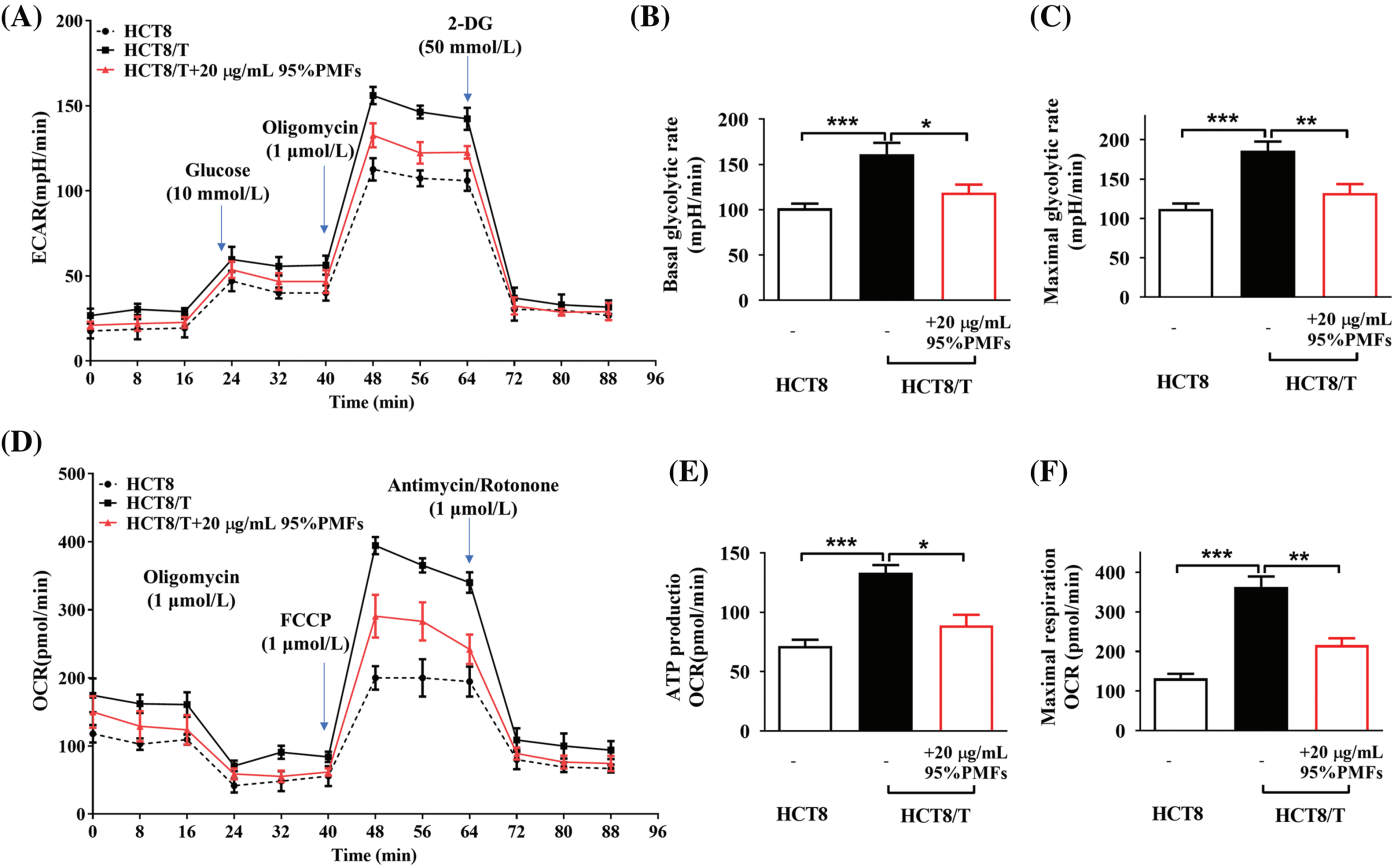
95%PMFs inhibit the aerobic glycolytic process in HCT8/T cells. (A) Effect of 95% PMFs on the extracellular acidification rate of HCT8/T cells; (B and C) basal cellular glycolysis, and maximum cellular glycolytic capacity obtained from the cell energy metabolic chart; (D) effect of 95% PMFs on the oxygen consumption rate of HCT8/T cells; (E and F) levels of cellular ATP production, and maximum cellular respiration obtained from the cell energy metabolic chart (*p < 0.05, **p < 0.01, ***p < 0.001).

To investigate whether PMFs reverse drug resistance by interfering with the aerobic glycolytic process in tumor cells, we measured key glycolytic parameters, including external acidification rate (ECAR) and oxygen consumption rate (OCR). The results indicate that compared to HCT8-sensitive cells, HCT8/T cells had a significantly higher external acidification rate, which decreased after treatment with 95% PMFs. HCT8/T cells have higher levels of baseline glycolytic capacity and maximal glycolytic capacity, and PMFs can inhibit the glycolytic capacity of HCT8/T cells (Figs. 4A–4C). Similarly, HCT8/T cells had a higher rate of oxygen consumption, as well as elevated levels of both ATP production and maximal respiration compared to sensitive cells. After treatment with PMFs, the respiration levels of HCT8/T cells were inhibited, as oxygen consumption rate, ATP production and maximal respiration levels decreased (Figs. 4D–4F). Therefore, PMFs may play a role in reversing drug resistance in tumor cells by inhibiting cellular aerobic glycolysis processes.

### The reversal of drug resistance by PMFs may be linked to the ROS-autophagy axis

**Figure 5:**
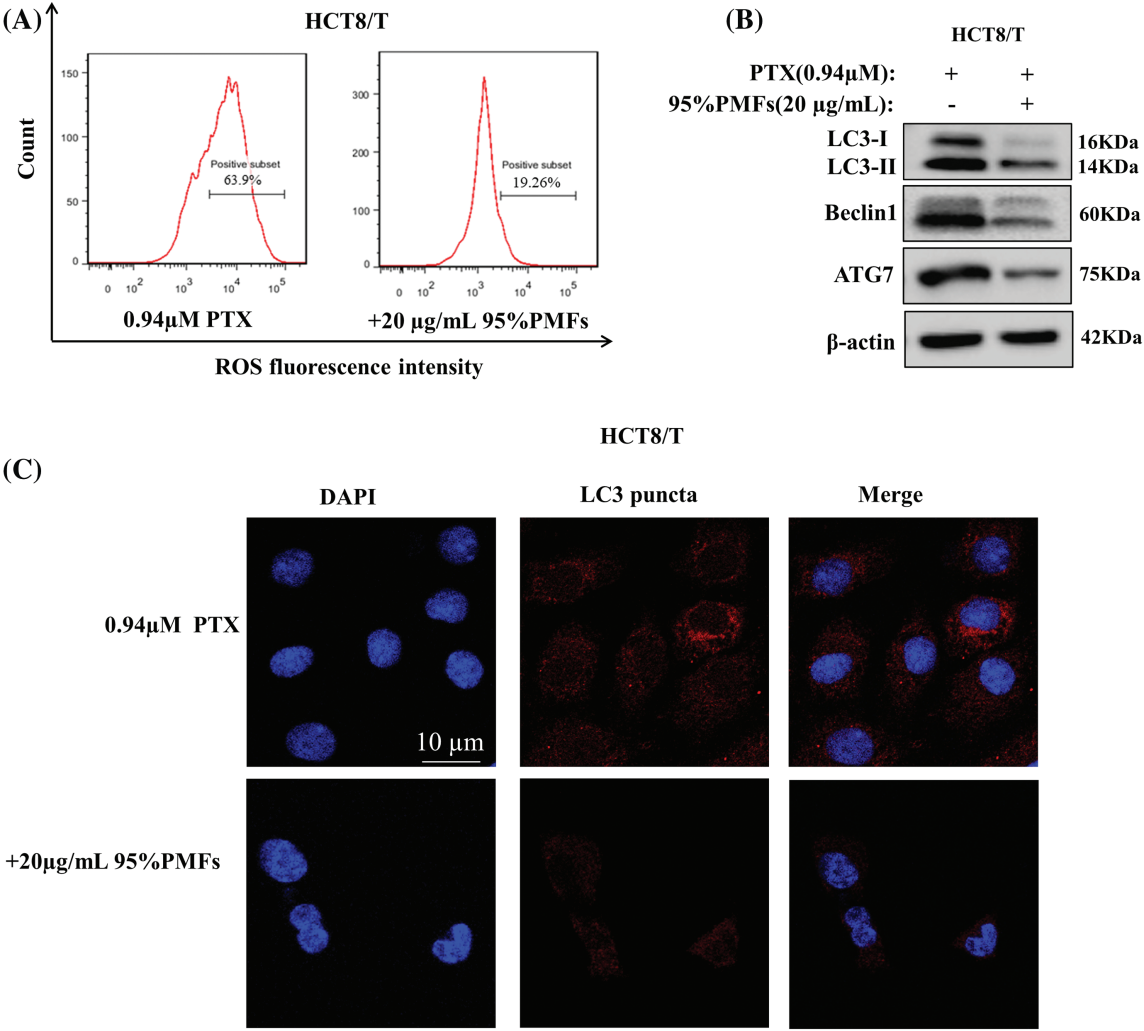
The reversal of drug resistance by PMFs may be associated with the ROS-autophagy axis. (A) Detection of ROS content by flow cytometry; (B) the expression of LC3-I, LC3-III, Beclin1, and ATG7 proteins in HCT8/T cells was analyzed by WB assays; (C) detection of autophagosomes by LeicaDM2500 fluorescence microscopy. Scale bar = 10 μm.

To investigate whether PMFs reverse drug resistance by mediating the ROS-autophagy signaling axis, we examined the changes in ROS content, autophagy-related protein expression levels, and the number of autophagosomes after the action of PMFs. Compared to PTX alone, ROS content was significantly reduced and autophagy-related proteins, including LC3-I, LC3-III, Beclin1, and ATG7 expression were down-regulated in HCT8/T cells treated with 95% PMFs for 48 h followed by PTX (Figs. 5A and 5B). Furthermore, we noticed that compared to HCT8/T cells treated with PTX alone, PMFs treatment for 24 h followed by PTX significantly reduced the number of intracellular autophagosomes (Fig. 5C). The above results demonstrated that PMFs may reverse drug resistance in HCT8/T cells by affecting the ROS-autophagy signaling axis.

## Discussion

Colon cancer is the second leading cause of cancer-related deaths worldwide. Despite ongoing efforts to reduce morbidity and mortality, drug resistance develops in almost all colon cancer patients, resulting in relapse for approximately 50% of patients [25]. The five-year relative survival rate for colon cancer remains low at 64.7% [26]. The developed drug resistance limits the therapeutic efficacy of chemotherapeutic agents for advanced colon cancer and metastatic colon cancer, ultimately leading to chemotherapy failure. Therefore, there is an urgent need for new drug-adjuvant chemotherapeutic agents in the clinic to overcome drug resistance in colon cancer.

Drug resistance in colon cancer is associated with several factors, with P-gp being recognized as the most crucial factor. P-gp, a member of the ABC transporter protein superfamily, is distributed in several vital organs, including the small intestine, liver, and kidney. Through ATP hydrolysis energy, it plays a critical role as a barrier to safeguard cells against toxic substances by regulating the efflux of both exogenous and endogenous substances [27]. Most of the anticancer drugs used in clinics act as P-gp substrates, such as paclitaxel, colchicine, 5-fluorouracil (5FU), and oxaliplatin [28]. As a result, overexpressed P-gp promotes the efflux of substrate anticancer drugs, leading to the development of tumor-acquired drug resistance (MDR) [29]. ROS and autophagy are also closely associated with drug resistance in colon cancer. ROS production and elimination are closely related to tumor cell redox homeostasis [30], and the dependence on redox homeostatic regulation is regarded as the main reason for the acquisition of drug resistance in tumors [31]. The use of chemotherapeutic agents disrupts the intracellular redox balance, leading to the accumulation of ROS, which promotes the expression of transporter proteins and induces the development of drug resistance in tumor cells [32]. And the increase in ROS may allow cancer cells that already have high levels of ROS to escape death [33]. Autophagy is regulated by the highly conserved autophagy-associated gene (ATG) gene and is induced mainly by inhibition of the mTOR signaling pathway during oxidative stress [34], which usually involves the formation of double membrane bodies called autophagosomes. The autophagy-associated proteins LC3-I and LC3-II are involved in lysosome formation through the ubiquitin-like pathway, and increased expression can promote autophagy [35]. Compared to normal tissues, LC3-II is overexpressed in advanced colon cancer cells, promoting autophagy in drug-resistant cells, and leading to increased invasion of colon cancer cells [36]. Autophagy plays a dual role in drug resistance and tumor progression [37]. However, inhibition of autophagy is believed to enhance the sensitivity of tumor cells to chemotherapy [38]. The findings of certain antitumor medications, specifically Vitexin, indicate that autophagy inhibition leads to the reversal of drug resistance in colon cancer [39]. Furthermore, research indicates that Reactive Oxygen Species (ROS) can trigger autophagy and modulate mTOR activity through different pathways [40]. Consequently, ROS and autophagy may jointly establish a ROS-autophagy signaling axis that controls drug resistance. The present study discovered that HCT8/T cells represented the characteristics of increased ROS amounts, enhanced expression of the autophagy-associated proteins LC3-I and LC3-II, and considerably higher PTX resistance in contrast to sensitive HCT8 cells. The increased resistance indexes indicated that HCT8/T cells have greater resistance to PTX, and the use of PTX alone in colon cancer therapy would lead to a reduction in drug effectiveness due to the emergence of MDR.

Polymethoxylated flavones (PMFs), a subgroup of plant flavonoids, display biological activity that inhibits tumor growth, invasion, and metastasis and enhances apoptosis [41]. PMFs mainly contain two components, nobiletin (5,6,7,8,3′,4′-hexamethoxyflavone), and tangeretin (5,6,7,8,4′-pentamethoxyflavone), of which nobiletin has the highest content [42]. Tangeretin is highly effective in inhibiting cancer cell growth [43], while nobiletin has no significant inhibitory effect [20,22]. Prior research has shown that specific PMF derivatives, such as 5-hydroxy polymethoxyflavones [43] and 5-demethylnobiletin [44], induce cell cycle arrest and facilitate apoptosis, thereby decreasing the proliferation of colon cancer cells. However, previous studies have focused primarily on the direct inhibition mechanism of PMFs against colon cancer rather than exploring their potential to sensitize drug-resistant cells to chemotherapeutic agents. Although PTX is not a standard treatment for colon cancer, it has shown significant promise in previous research. During the small-scale treatment of patients with colon cancer using PTX, treatment with PTX significantly reduced the production of tumor markers and improved the overall survival rate of the patients [45]. Therefore, in contrast to previous studies, we focused on the role of PMFs in reversing PTX resistance in colon cancer. Our study revealed that 95% of PMFs displayed the highest effectiveness and PMFs were able to reverse drug resistance in colon cancer in a concentration-dependent manner. Additionally, PMFs demonstrated an increase in the sensitivity of tumors to PTX and inhibited the growth of MDR tumors in the HCT8/T xenograft nude mouse model *in vitro* experiments. Furthermore, a decrease in both tumor volume and weight was observed to some extent. Therefore, PMFs could reverse colon cancer resistance and improve PTX efficacy. Moreover, colon cancer cells were found to have inhibitory effects by PMFs on ROS and autophagy. Combined with PTX treatment, PMFs significantly decreased the number of autophagosomes, reduced the ROS content of HCT8/T cells, and down- regulated autophagy-related proteins LC3-I, LC3-II, Beclin1, and ATG7 expression levels. The findings demonstrated that PMFs have the potential to overcome drug resistance in colon cancer through the ROS-autophagy signaling axis. However, additional research is still needed to explore the mechanism involved.

The Warburg effect, first discovered in 1924 by Otto Warburg, describes the correlation between the acquisition of ATP by tumor cells and the process of aerobic glycolysis. Tumor cells utilize a specific pathway for breaking down glucose to produce energy through aerobic glycolysis even in an oxygen-rich environment, distinguishing them from normal tissues that prefer Oxidative phosphorylation (OXPHOS). Although aerobic glycolysis produces less energy, cancer cells have significantly higher ATP levels than normal cells [46]. In colon cancer cell lines, ATP levels in cancer cells are twice as high as those in sensitive parental cell lines [47]. Energy reserves play a critical role in drug-resistant cells, and elevated ATP levels are necessary for drug-resistant cancer cells [48]. Our results demonstrated that the combination of PMFs and PTX significantly reduced both ECAR and OCR, and inhibited cellular ATP production and maximal respiration compared to PTX treatment alone. Therefore, PMFs were able to reverse PTX resistance by interfering with the aerobic glycolysis process in HCT8/T cells.

In addition, previous studies indicate that the Warburg effect is closely associated with the ROS-autophagy signaling axis [49], and they may both contribute to the formation of the aerobic glycolysis-ROS-autophagy signaling axis, which together mediate the reversal of tumor drug resistance. Cellular metabolism and ROS are intricately connected. Quijano et al. demonstrated that cellular metabolism affects ROS production and redox homeostasis, while on the other hand, ROS can also affect the energy metabolism of tumor cells by regulating key enzymes in the metabolic process [50]. In addition, cellular metabolism has a close relationship with autophagy, as Zhang et al. found that autophagy can promote excessive tumor proliferation by providing amino acids and other essential molecules when macromolecular raw materials are scarce [51]. Therefore, tumor cell metabolism, ROS production, and autophagy may have interconnected signaling pathways that jointly contribute to the emergence of tumor drug resistance. However, there are still some shortcomings in this study, such as the lack of P-gp transport test to further evaluate the differences in P-gp function after drug administration. We believed there are other mechanisms for the sensitizing effects of PMFs which will be explored in the future. Our present work should expedite the exploration and use of PMFs in enhancing the efficacy of chemotherapeutic agents in experimental animal studies as well as clinical trials.

## Conclusion

**Figure 6:**
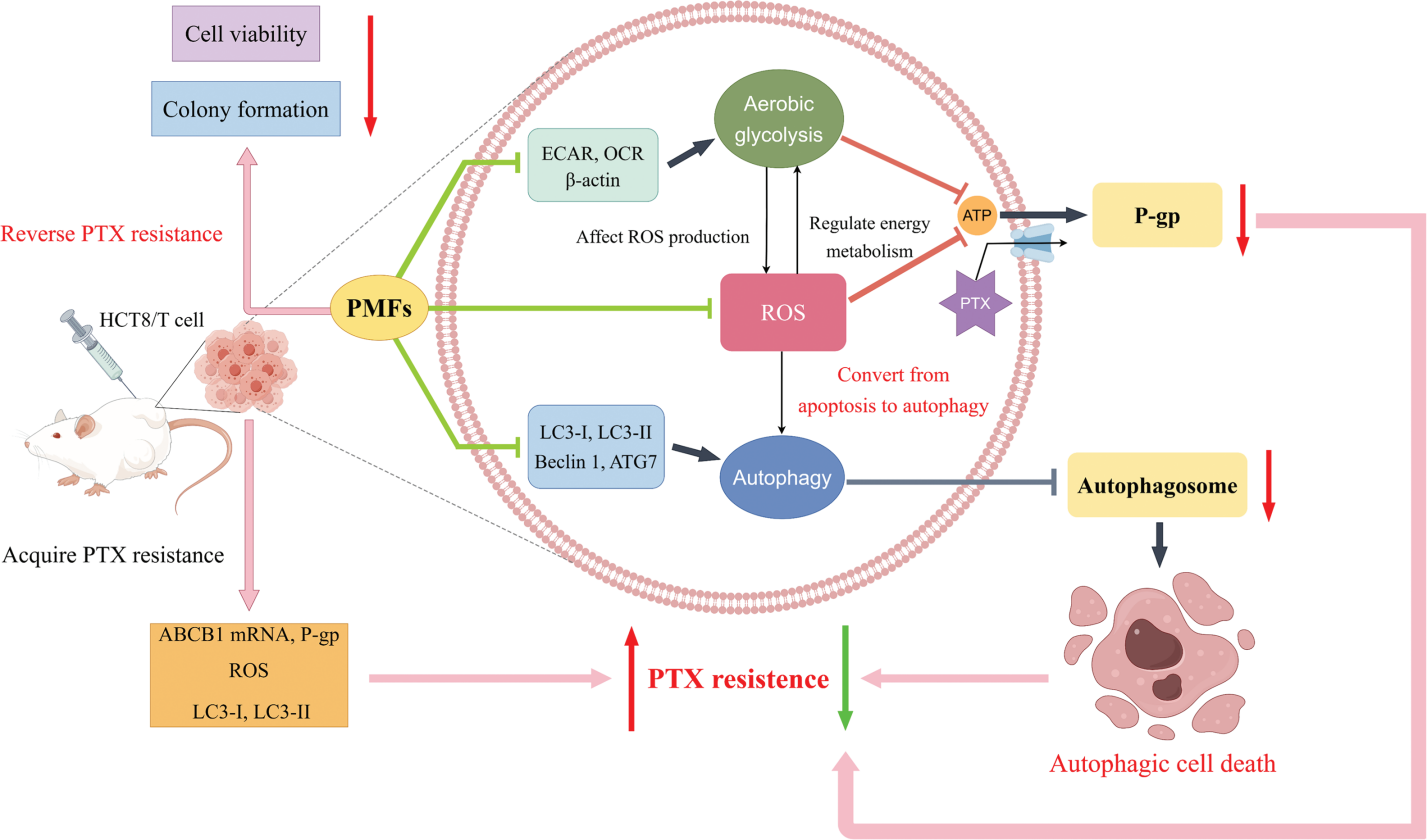
Schematic diagram of the study design. The high resistance of HCT8/T cells was confirmed through the examination of ABCB1, P-gp, ROS, and other indicators. Upon administration of PTX on HCT8/T cells, the extracellular acidification rate (ECAR), oxygen consumption rate (OCR), and ROS content increased, while autophagy-related proteins and autophagosomes were upregulated. When 95% PMFs were combined with PTX, the ECAR, OCR, and ROS levels decreased, and autophagy-related proteins and autophagosomes were downregulated. *In vitro* experiments have demonstrated a decrease in cell viability and cell colony formation. *In vivo* experiments have shown that tumor formation is suppressed. These findings mutually corroborate that 95% PMFs can effectively reverse drug resistance in colon cancer (Schematic diagram was drawn by Figdraw).

In summary, our research indicates that HCT8/T cells possess a heightened state of drug resistance, the resistance indicators including ABCB1 mRNA, P-gp, ROS, and autophagy-related proteins LC3-I and LC3-II showed significant elevation in HCT8/T cells. Furthermore, PMFs were able to effectively reverse drug resistance in colon cancer by reducing ROS and autophagosome production. The potential mechanism may involve the aerobic glycolysis-ROS-autophagy signaling axis (Fig. 6).

In our future studies, we will further investigate whether PMFs can reverse drug resistance by affecting the aerobic glycolysis-ROS-autophagy signaling axis based on the results of the present experiments, and explore the potential relationship between PMFs’ reversal of tumor drug resistance by inhibiting P-gp and the aerobic glycolysis-ROS-autophagy signaling axis. To promote the clinical application of nobiletin, studies in the mechanism of reversal of drug resistance are urgently needed.

## Data Availability

The data that support the findings of this study are available from the corresponding author, upon reasonable request.
